# Antimicrobial Susceptibility and Molecular Characterization Using Whole-Genome Sequencing of Clostridioides difficile Collected in 82 Hospitals in Japan between 2014 and 2016

**DOI:** 10.1128/AAC.01259-19

**Published:** 2019-11-21

**Authors:** Kotaro Aoki, Shinobu Takeda, Takashi Miki, Yoshikazu Ishii, Kazuhiro Tateda

**Affiliations:** a Department of Microbiology and Infectious Diseases, Toho University School of Medicine, Tokyo, Japan; b Drug Discovery Research, Astellas Pharma, Inc., Tokyo, Japan; c Japan Astellas Pharma, Inc., Tokyo, Japan

**Keywords:** *Clostridioides difficile*, whole-genome sequencing, fidaxomicin

## Abstract

We studied the antimicrobial susceptibility and molecular characteristics, using draft whole-genome sequencing, of Clostridioides (Clostridium) difficile strains before and after treatment in adults with C. difficile infection (CDI) enrolled in a phase III, randomized, nationwide study of fidaxomicin versus vancomycin in Japan (ClinicalTrials.gov identifier NCT02179658). C. difficile strains were cultured from stool samples collected before and after standard treatment with either fidaxomicin or vancomycin. Overall, 285 C. difficile strains were recovered, with 188 derived from CDI cases at baseline (87 patients received fidaxomicin, and 101 received vancomycin). No strains isolated from episodes of CDI at baseline were shown to have reduced susceptibilities to fidaxomicin (MIC, ≥1 mg/liter) or resistance to vancomycin and metronidazole. Thirty-three sequence types (STs) were identified, the most common being ST17 (*n* = 61 [32.4%]), ST8 (*n* = 26 [13.8%]), and ST2 (*n* = 21 [11.2%]). Core-genome single-nucleotide polymorphism analysis showed that outbreaks of C. difficile were unlikely to have occurred at each hospital. The predominant toxin gene profile was *tcdA^+^ tcdB^+^ cdtA-cdtB*^−^ (*n* = 149 [79.3%]). Six of 87 patients who received fidaxomicin harbored C. difficile isolates with reduced fidaxomicin susceptibilities conferred by previously described mutations, Val1143Leu/Gly/Asp in RpoB or Arg89Gly in RpoC or putative mutations, Gln1149Pro in RpoB, or Arg326Cys in RpoC. Allelic exchange studies of these putative mutations were not performed. Prior to fidaxomicin use, we found no C. difficile strains with reduced fidaxomicin susceptibility causing CDI in Japan; however, mutant strains with reduced fidaxomicin susceptibility were detected after fidaxomicin treatment.

## Abstract (precis)

We studied the antimicrobial susceptibility and molecular characteristics, using draft whole-genome sequencing, of Clostridioides (Clostridium) difficile strains before and after treatment in adults with C. difficile infection (CDI) enrolled in a phase III, randomized, nationwide study of fidaxomicin versus vancomycin in Japan (ClinicalTrials.gov identifier NCT02179658). C. difficile strains were cultured from stool samples collected before and after standard treatment with either fidaxomicin or vancomycin.

## INTRODUCTION

Clostridioides (Clostridium) difficile is a prominent cause of antimicrobial-associated diarrhea (1). Recurrent C. difficile infection (CDI) occurs in 20 to 30% of cases (2, 3) and is associated with significant morbidity, mortality, and excess economic health care burden (4, 5). Fidaxomicin (FDX), a narrow-spectrum antibiotic, is associated with improved sustained clinical cure and reduced recurrence of CDI compared with vancomycin (VCM) (6, 7).

Whole-genome sequencing of C. difficile provides a valuable tool for profiling strains and evaluating their genetic diversity, enabling assessments of the epidemiology of strains implicated in infection recurrences and outbreaks (8). Data generated from whole-genome sequencing are of particular relevance to evaluate the presence of genes and genetic mutations linked to antibiotic resistance. Reduced susceptibility to FDX among C. difficile strains has been associated with single, nonsynonymous mutations of genes encoding RNA polymerase subunits β (RpoB) and βʹ (RpoC) (9–11). Only one strain isolated from a patient cured of CDI had an elevated FDX MIC of 16 mg/liter at the time of recurrence in two phase III randomized, double-blind trials at sites in North America and seven European countries (12). This strain’s mechanism of reduced susceptibility to FDX was not analyzed.

Few studies have evaluated clinically relevant strains of C. difficile in Japan with a view to examine their antibiotic susceptibilities and resistance mechanisms. Such information may help tailor the choice of treatment for CDI. In this study, we performed molecular characterization using whole-genome sequencing of C. difficile strains isolated from 188 samples from patients with CDI enrolled in a phase III study in Japan.

## RESULTS

### Participants and C. difficile strains.

**FIG 1:**
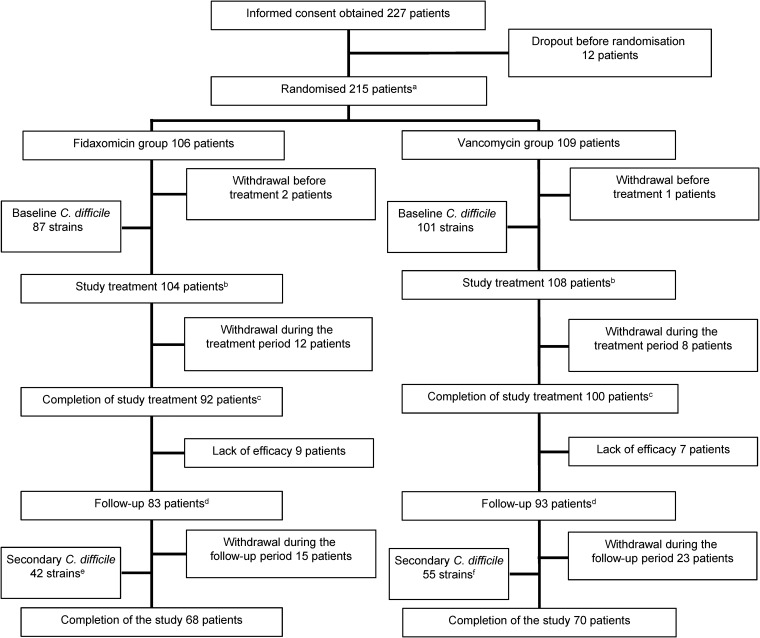
Patient flow in the phase III, randomized, nationwide study of fidaxomicin versus vancomycin in Japan. a, patients diagnosed with Clostridioides difficile infection (CDI) were positive for C. difficile toxin A, B, or both in a stool specimen obtained within 96 h before randomization. b, days 1 to 10. c, if a stool specimen was obtained from the patient within 24 h of completing the treatment (days 10 and 11), C. difficile culture was performed. d, if diarrhea occurred within 28 days (± 3 days) after treatment, C. difficile culture was performed. e, one secondary C. difficile strain was isolated unexpectedly during fidaxomicin administration. f, two secondary C. difficile strains were isolated unexpectedly during vancomycin administration.

In all, 215 participants were randomized in the phase III study, with 106 participants to FDX and 109 participants to VCM (Fig. 1). A total of 285 C. difficile strains were recovered from the stool samples of 188 patients (87/106 [82.1%] who received FDX and 101/109 [92.7%] who received VCM). Of these, 188 were nonduplicate C. difficile strains recovered from patients with CDI at baseline (before receiving FDX or VCM; Fig. 1). Secondary strains were recovered from 42/87 (48.3%) patients after receiving FDX and 55/101 (54.5%) patients after receiving VCM (Fig. 1).

### Whole-genome sequence analysis of C. difficile strains.

We performed whole-genome sequencing of all 285 C. difficile isolates, with a mean (standard deviation) map read depth of 76.7 (30.0) (see Data Set S1 in the supplemental material). The assembled genomes had an average of 218.7 (128.6) contigs and an *N*_50_ value of 53,033 bp (39,128 bp).

### Multilocus sequence typing, phylogenetic analysis, and toxin gene patterns.

**FIG 2:**
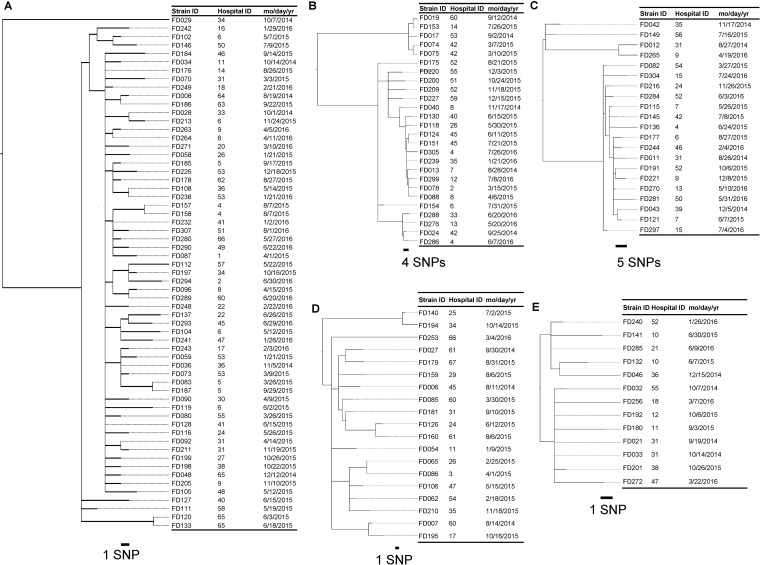
Phylogenetic tree of Clostridioides difficile strains belonging to each sequence type (ST) constructed with maximum likelihood phylogenetic analysis based on single-nucleotide polymorphisms (SNPs) in the core genome, excluding estimated homologous recombination. The scale of the distances corresponds to the average number of substitutions per site and SNP. (A) Phylogenetic tree of 61 strains of C. difficile ST17. A core genome region, amounting to 58.4% (2,505,609/4,290,252 bp), was shared with the genome of a reference strain, C. difficile 630 (ST54). (B) Phylogenetic tree of 26 strains of C. difficile ST8. A core genome region, amounting to 32.0% (1,374,263/4,290,252 bp), was shared with the genome of a reference strain, C. difficile 630 (ST54). (C) Phylogenetic tree of 22 strains of C. difficile ST2. A core genome region, amounting to 34.1% (1,461,002/4,290,252 bp), was shared with the genome of a reference strain, C. difficile 630 (ST54). (D) Phylogenetic tree of 19 strains of C. difficile ST81. A core genome region, amounting to 72.6% (3,114,637/4,290,252 bp), was shared with the genome of a reference strain, C. difficile 630 (ST54). (E) Phylogenetic tree of 13 strains of C. difficile ST183. A core genome region, amounting to 41.8% (1,795,356/4,290,252 bp), was shared with the genome of a reference strain, C. difficile 630 (ST54).

Using multilocus sequence typing (MLST), 188 C. difficile strains isolated before treatment were classified into 33 sequence types (STs) (Table 1). ST470 was a type not previously detected. Strains belonging to ST17 were the most common, accounting for 32.4% (*n* = 61) of all isolates from CDI cases at baseline, followed by those belonging to ST8 (*n* = 26 [13.8%]), ST2 (*n* = 21 [11.2%]), ST81 (*n* = 19 [10.1%]), and ST183 (*n* = 13 [6.9%]) (Table 1). No single ST was found to accumulate in one center. Core-genome single-nucleotide polymorphism analysis suggested that outbreaks of C. difficile were unlikely to have occurred at each hospital (Table 2 and Fig. 2). The nucleotide substitution rate of C. difficile ST17, as representative of C. difficile, was estimated based on draft whole-genome sequence data. The average substitution in the core genome was estimated to be 5.2 single-nucleotide polymorphisms (SNPs)/core genome/year (95% highest posterior density interval, 0.1, 15.3).

**TABLE 1:** Multilocus sequence typing and estimated PCR ribotype of Clostridioides difficile strains recovered from patients with C. difficile infection

Sequence type	Clonal complex^*a*^	MLST clade^*b*^	Estimated RT^*c*^	No. (%) of strains	No. of centers	No. of strains with antibiotic treatment of:	
						Fidaxomicin	Vancomycin
ST17	CC3	1	RT018	61 (32.4)	43	28	33
ST8	CC3	1	RT002	26 (13.8)	20	12	14
ST2	CC3	1	RT014/020/076/220	21 (11.2)	10	5	16
ST81	CC37	4	NA	19 (10.1)	17	9	10
ST183	CC3	1	NA	13 (6.9)	13	6	7
ST55	CC99	1	NA	5 (2.7)	5	5	0
ST37	CC37	4	RT017	4 (2.1)	4	3	1
ST5	CC5	3	RT023/063	3 (1.6)	3	2	1
ST14	CC3	1	RT014	3 (1.6)	3	0	3
ST15	CC3	1	RT010	3 (1.6)	3	1	2
ST103	CC103	1	NA	3 (1.6)	3	0	3
ST3	CC3	1	RT001/009/072/220	2 (1.1)	2	1	1
ST27	CC3	1	RT067	2 (1.1)	2	0	2
ST48	CC3	1	NA	2 (1.1)	2	2	0
ST58	CC3	1	NA	2 (1.1)	2	1	1
ST109	CC238	4	NA	2 (1.1)	2	1	1
ST1	CC3	2	RT027	1 (0.5)	1	1	0
ST6	CC3	1	RT005	1 (0.5)	1	0	1
ST11	CC11	5	RT078	1 (0.5)	1	1	0
ST13	CC3	1	RT129	1 (0.5)	1	1	0
ST26	CC3	1	RT039/140	1 (0.5)	2	0	1
ST35	CC3	1	RT046	1 (0.5)	1	0	1
ST42	CC3	1	RT106/118/174	1 (0.5)	1	1	0
ST47	CC3	2	NA	1 (0.5)	1	0	1
ST53	CC3	1	RT103	1 (0.5)	1	1	0
ST54	CC3	1	RT012	1 (0.5)	1	1	0
ST59	ND	1	NA	1 (0.5)	1	1	0
ST67	CC3	2	RT019	1 (0.5)	1	0	1
ST82	CC3	1	NA	1 (0.5)	1	0	1
ST98	CC3	1	NA	1 (0.5)	1	1	0
ST100	ND	1	NA	1 (0.5)	1	1	0
ST182	CC3	1	NA	1 (0.5)	1	1	0
ST470	CC3	1	NA	1 (0.5)	1	1	0
Total				188		87	101

a ND, not assigned to any CC.

b According to the PubMLST website (https://pubmlst.org/cdifficile/).

c NA, not assigned, as information on the relationship between ST and ribotype (RT) was not available.

**TABLE 2:** Toxin-encoding gene profiles of the Clostridioides difficile strains isolated from C. difficile infection episodes at baseline

ST	No. of strains	Toxin gene pattern^*a*^			
		*tcdA*^+^ *tcdB*^+^ *cdtA-cdtB*^−^	*tcdA*^−^ *tcdB*^+^ *cdtA-cdtB*^−^	*tcdA*^+^ *tcdB*^+^ *cdtA-cdtB^+^*	Nontoxigenic
ST17	61	61	0	0	0
ST8	26	26	0	0	0
ST2	21	21	0	0	0
ST81	19	0	19	0	0
ST183	13	13	0	0	0
ST55	5	5	0	0	0
ST37	4	0	4	0	0
ST5	3	0	0	3	0
ST14	3	3	0	0	0
ST15	3	0	0	0	3
ST103	3	3	0	0	0
Other^*b*^	27	17	0	2	8
Total	188	149	23	5	11

a *tcdA,* encoding toxin A; *tcdB,* encoding toxin B; *cdtA,* encoding binary toxin A; *cdtB,* encoding binary toxin B.

b Other indicates STs with fewer than two assigned strains, as follows: ST1, ST3, ST6, ST11, ST13, ST26, ST27, ST35, ST42, ST47, ST48, ST53, ST54, ST58, ST59, ST67, ST82, ST98, ST100, ST103, ST109, ST182, ST188, ST223, and ST470.

The toxin-encoding gene profiles of each ST are included in Table 2. ST and toxin gene profiles, excluding *tcdC*, were consistent. *tcdA*^+^ *tcdB*^+^ *cdtA-cdtB*^−^ was the dominant profile (*n* = 149 [79.3%]), followed by *tcdA*^−^ *tcdB*^+^ *cdtA-cdtB*^−^ (*n* = 23 [12.2%]) and *tcdA*^+^ *tcdB*^+^ *cdtA-cdtB*^+^ (*n* = 5 [2.7%]). Eleven (5.9%) nontoxigenic strains were recovered. The nucleotide deletion in *tcdC* at position 10 to a frameshift mutation was found in one of three ST5 strains. The nucleotide deletion in *tcdC* at position 117 to a frameshift mutation was found in one ST1 strain. A nonsense nucleotide mutation (from cytosine to thymine) at nucleotide position 184 was found in two ST5 strains and one ST11 strain. The amino acid insertion of lysine-alanine-glycine-glycine-alanine-lysine at position 114 in TcdC was detected in one strain each belonging to ST8, ST17, ST47, ST54, and ST82.

### Antibiotic susceptibilities of C. difficile strains isolated from CDI episodes at baseline.

No strains having reduced susceptibilities to FDX (MIC, ≥1 mg/liter) or with resistance to VCM and metronidazole (MNZ) were isolated from episodes of CDI at baseline. All strains belonging to ST17, ST81, and ST183 showed resistance to moxifloxacin (MFLX) and clindamycin (CLDM) (Table 3). The numbers of strains having resistance to MFLX and CLDM among ST8 were 18 (69.2%) and 21 (80.8%) of 26 strains, respectively, and among ST2, the respective numbers of strains were 3 (14.3%) and 18 (85.7%) of 21 strains. One ST1 strain was resistant to MFLX (Table 3).

**TABLE 3:** Antibiotic susceptibilities of the Clostridioides difficile strains isolated from C. difficile infection episodes at baseline

ST	No. of strains	Resistance data by antibiotic treatment^*a*^																			
		Fidaxomicin				Vancomycin				Metronidazole				Moxifloxacin				Clindamycin			
		MIC data (mg/liter)			No. (%) of resistant strains	MIC data (mg/liter)			No. (%) of resistant strains	MIC data (mg/liter)			No. (%) of resistant strains	MIC data (mg/liter)			No. (%) of resistant strains	MIC data (mg/liter)			No. (%) of resistant strains
		Range	MIC_50_	MIC_90_		Range	MIC_50_	MIC_90_		Range	MIC_50_	MIC_90_		Range	MIC_50_	MIC_90_		Range	MIC_50_	MIC_90_	
17	61	0.008 to 0.12	0.03	0.12	ND	0.25 to 2	0.25	1	0	0.12 to 2	0.5	1	0	16 to >128	32	32	61 (100)	64 to >128	>128	>128	61 (100)
8	26	0.015 to 0.12	0.03	0.12	ND	0.25 to 1	0.5	0.5	0	0.12 to 1	0.5	1	0	1 to >128	32	32	18 (69.2)	1 to >128	8	>128	21 (80.8)
2	21	0.03 to 0.5	0.06	0.25	ND	0.25 to 1	0.5	0.5	0	0.25 to 1	0.5	1	0	1 to 32	2	8	3 (14.3)	2 to 16	16	16	18 (85.7)
81	19	0.015 to 0.25	0.12	0.25	ND	0.25 to 0.5	0.5	0.5	0	0.12 to 1	0.5	1	0	16 to >128	16	>128	19 (100)	16 to >128	>128	>128	19 (100)
183	13	0.015 to 0.12	0.06	0.12	ND	0.25 to 1	0.5	0.5	0	0.25 to 1	0.5	0.5	0	>128	>128	>128	13 (100)	64 to >128	128	>128	13 (100)
55	5	0.008 to 0.06	NA	NA	ND	0.25 to 0.5	NA	NA	NA	0.12 to 0.5	NA	NA	NA	1 to 32	NA	NA	2 (NA)	4 to >128	NA	NA	3 (NA)
37	4	0.015 to 0.25	NA	NA	ND	0.5	NA	NA	NA	0.25 to 0.5	NA	NA	NA	2	NA	NA	0 (NA)	64 to >128	NA	NA	4 (NA)
5	3	≤0.004 to 0.12	NA	NA	ND	0.5	NA	NA	NA	0.25 to 1	NA	NA	NA	2 to 8	NA	NA	1 (NA)	4 to 16	NA	NA	2 (NA)
14	3	0.06 to 0.25	NA	NA	ND	0.25 to 0.5	NA	NA	NA	0.25 to 1	NA	NA	NA	2	NA	NA	0 (NA)	8 to 16	NA	NA	3 (NA)
15	3	0.015 to 0.03	NA	NA	ND	0.5	NA	NA	NA	0.25 to 1	NA	NA	NA	2 to 16	NA	NA	2 (NA)	2 to >128	NA	NA	2 (NA)
Other^*b*^	30	≤0.004 to 0.25	0.06	0.12	ND	0.25 to 1	0.5	1	0	0.12 to 2	0.5	1	0	1 to 32	2	16	5 (16.7)	1 to >128	16	>128	26 (86.7)
Total	188	≤0.004 to 0.5	0.06	0.12	ND	0.25 to 2	0.5	0.5	0	0.12 to 2	0.5	1	0	1 to >128	32	>128	124 (66.0)	1 to >128	128	>128	172 (91.5)

a NA, not applicable (owing to the number of strains being too small); ND, not determined.

b Other, STs with fewer than two assigned strains, as follows: ST1, ST3, ST6, ST11, ST13, ST26, ST27, ST35, ST42, ST47, ST48, ST53, ST54, ST58, ST59, ST67, ST82, ST98, ST100, ST103, ST109, ST182, ST188, ST223, and ST470.

### Antibiotic resistance determinants of C. difficile strains.

The amino acid substitutions Gly91Asp, Ser94Ile, and Pro115Ser in RNA polymerase subunit α (RpoA) were detected in seven C. difficile strains. Asp492Val, His502Asn, Arg505Lys, Ile548Met, Ile750Met/Val, Glu1037Gln, Asp1160Glu, Ala1205Val, Asn1207Ala, Ala1208Thr, and Asp1232Glu in RNA polymerase subunit β (RpoB) were detected in 68 C. difficile strains. Thr543Ile, Asn564Lys, Ala617Ser, Ile788Val, Ile833Leu, and Pro1084Thr in RNA polymerase subunit βʹ (RpoC) were detected in 10 C. difficile strains. Furthermore, C. difficile strains in which any RpoA, RpoB, or RpoC amino acid mutation was detected showed no reduced susceptibility to FDX (Table S1). Of 124 MFLX-resistant C. difficile strains, 117 strains had amino acid substitutions from threonine to isoleucine or valine at GyrA position 82 (GyrA Thr82Ile/Val) or from aspartate to alanine, asparagine, or valine at GyrB position 426 (GyrB Asp426Ala/Asn/Val) (Tables 3 and S1). All strains belonging to ST17, ST81, and ST183 had GyrA Thr82Ile/Val or GyrB Asp426Ala/Asn/Val substitutions, while few strains belonging to other STs, except ST8, had these substitutions. Of 172 CLDM-resistant strains, 67 were carrying *erm*(B). No strains showing CLDM susceptibility had *erm*(B).

### Antibiotic susceptibilities and mutational analysis of C. difficile strains isolated before and after antibiotic treatment.

Of 188 CDI cases, C. difficile strains were recovered from the stool samples of 97 patients at the follow-up appointment within 4 weeks after treatment with FDX (*n* = 42) or VCM (*n* = 55; Fig. 1). Paired C. difficile strains isolated in 78 of 97 cases belonged to the same ST. ST17 was the most common type, occurring in 32.1% (*n* = 25) of patients, followed by strains belonging to ST2 (*n* = 12), ST8 (*n* = 11), ST183 (*n* = 6), and ST81 (*n* = 5). In seven cases in the FDX treatment group and 12 cases in the VCM group, strains isolated at baseline and during a secondary episode of CDI belonged to different STs.

The FDX, MNZ, and MFLX MICs had increased by more than 4-fold compared with the MICs before treatment in six cases, three cases, and one case, respectively (Table 4). C. difficile strains with substantially reduced susceptibility to FDX were obtained only in the patient group that had received FDX treatment (six isolates with 30- to 2,000-fold reduced susceptibility) and not from the VCM group. No isolate from the VCM group showed reduced VCM susceptibility (Table 4). There was no substantial increase in VCM and CLDM MICs. Of the three cases from whom the isolated C. difficile strains had 4-fold reduced susceptibility to MNZ after VCM treatment, only one case (patient no. 8) had received MNZ prior to VCM treatment. One C. difficile isolate with 4-fold reduced susceptibility to MFLX was isolated from patient no. 157, who had not received any fluoroquinolone prior to VCM treatment. Six of 10 paired strains had reduced susceptibility to FDX after FDX treatment (MICs range, 0.25 to 64 mg/liter); all six patients who harbored these strains were reported to be cured after treatment.

**TABLE 4:** SNP analysis and antibiotic susceptibilities of 10 paired strains of Clostridioides difficile with MIC increases of more than 4-fold for any antibiotic tested^*a*^

Mutation category associated with decreased FDX sensitivity	Patient no.	Antibiotic treatment	Strain ID (isolation date [mo/day/yr])		MLST	Gene-encoded protein	SNP (amino acid substitution)	MIC of strain isolated before/after FDX or VCM treatment (mg/liter) for antibiotic^*b*^:				
			Before treatment	After treatment				FDX	VCM	MNZ	MFLX	CLDM
Already described	80	FDX	FD070 (3/3/2015)	FD077 (3/16/2015)	ST17	RpoB	G3427C (Val1143Leu)	**0.12/8**	0.25/0.25	0.5/0.5	32/16	>128/>128
	92	FDX	FD010 (8/21/2014)	FD020 (9/15/2014)	ST81	RpoC	A265G (Arg89Gly)	**0.12/4**	0.25/0.5	0.5/0.5	16/16	128/128
	155	FDX	FD128 (6/15/2015)	FD152 (7/20/2015)	ST17	RpoB	T3428G (Val1143Gly)	**0.03/16**	0.25/0.5	0.25/0.12	32/16	>128/>128
	194	FDX	FD282 (6/1/2016)	FD292 (6/27/2016)	ST8	RpoB	T3428A (Val1143Asp)	**0.06/>64**	0.5/1	0.5/0.12	32/32	16/16
	174	FDX	FD105 (5/12/2015)	FD131 (6/16/2015)	ST17	RpoC	A260G (Arg89Gly)	**0.03/64**	0.25/0.5	1/0.25	32/16	>128/>128
Putative						RpoC	C976T (Arg326Cys)					
	7	FDX	FD199 (10/26/2015)	FD219 (11/30/2015)	ST17	RpoB	A3446C (Gln1149Pro)	**0.03/0.25**	0.5/0.25	0.25/0.25	32/32	>128/>128
None	8	VCM	FD268 (5/9/2016)	FD279 (5/29/2016)	ST103	ND	ND	0.03/0.03	0.5/1	**0.25/1**	32/32	8/8
	33	VCM	FD265 (4/19/2016)	FD277 (5/24/2016)	ST2	ND	ND	0.12/0.06	0.5/1	**0.25/1**	2/2	16/16
	74	VCM	FD159 (8/6/2015)	FD183 (9/17/2015)	ST81	ND	ND	0.12/0.06	0.25/0.5	**0.12/0.5**	16/16	>128/>128
	157	VCM	FD094 (4/14/2015)	FD109 (5/19/2015)	ST67	ND	ND	0.12/0.06	0.5/0.5	1/1	**2/8**	16/16

a The 10 C. difficile infection cases shown were deemed to be clinically cured with fidaxomicin (FDX) or vancomycin (VCM) treatment. MLST, multilocus sequence type; ND, not determined; RpoB, DNA-directed RNA polymerase subunit β encoded by *rpoB*; RpoC, DNA-directed RNA polymerase subunit βʹ encoded by *rpoC*; SNP, single-nucleotide polymorphism.

b C. difficile isolate MICs shown in bold are those for which the MIC increased 4-fold or more between pre- and posttreatment with FDX or VCM. MNZ, metronidazole; MFLX, moxifloxacin; CLDM, clindamycin.

Identified single-nucleotide polymorphisms (SNPs) with amino acid substitutions were from valine to leucine, glycine, or aspartate at position 1143 in RpoB (Val1143Leu/Gly/Asp) and from arginine to glycine at position 89 (Arg89Gly) in RpoC, which are previously described mutations associated with decreased FDX sensitivity (Table 4). Amino acid substitutions from glutamine to proline at position 1149 in RpoB (Glu1149Pro) and from arginine to cysteine at position 326 in RpoC (Arg326Cys) were putative mutations (Table 4). No other SNPs were detected in the C. difficile strains with reduced susceptibility to FDX. SNPs were not detected in paired strains with reduced susceptibility to MNZ or MFLX (Table 4).

## DISCUSSION

In a previous study, ST17 (ribotype 018 [RT018]/smz) was the most common type isolated (frequency, 21.5% to 61.6%) from patients with CDI between 1999 and 2013 in Japan (13–18). ST17 was also the most predominant isolate in the present study, conducted from 2014 to 2016 in Japan, suggesting that the molecular epidemiology of C. difficile strains causing infections in Japan was relatively unchanged over time. Additionally, ST17 was a frequently isolated lineage implicated in CDI in South Korea and Italy (19–22); however, the genetic relationship to ST17 in Japan is unknown. Strains belonging to ST8 (RT002; detection frequency, 13.8%) and ST2 (RT014; detection frequency, 11.2%) were also common in our study, as RT002 was detected at a frequency of 4 to 6%, and RT014 was detected at a frequency of around 10% in European countries (23). ST81 (detection frequency, 10.1%) was toxin A negative, toxin B positive (A^−^ B^+^) and belonged to MLST clade 4, along with ST37 (RT017); these are dominant lineages in China and South Africa (17, 24–27).

Regarding C. difficile ST17 strains isolated in this study, there were nine pairs of strains that had a genetic distance of ≦2 SNPs, and while the possibility of a small number of transmission events could not be ruled out, there was no evidence of infection outbreaks (Fig. 2A). The transmission cutoff value based on the nucleotide substitution rate of C. difficile ST17 estimated in this study was evaluated as acceptable. The threshold of ≦2 SNPs was also adopted as the direct transmission cutoff value for STs other than ST17 (RT037) in previous reports (28, 29). For ST2, ST8, ST81, and ST183, when the transmission threshold of ≦2 SNPs was applied, no outbreak was reported involving any of these STs.

After antibiotic susceptibility testing of isolated C. difficile strains, SNP analysis of whole-genome sequences from the strains with reduced susceptibilities isolated before and after treatment with FDX or VCM suggested that there was reduced susceptibility to FDX in six strains after FDX treatment. Mutations at positions 1143 of RpoB (Val1143Leu/Gly/Asp) and 89 of RpoC (Arg89Gly) have been reported to be determinants of reduced susceptibility to FDX (9). RpoB Gln1149Pro and RpoC Arg326Cys mutations had not been reported in previous studies. In a previous *in vitro* study, the frequency of detection of C. difficile strains with reduced susceptibility to FDX at 4-fold the MIC ranged from 1.28 × 10^−8^ to <1.41 × 10^−9^ (30). Also, the Val1143Gly/Asp mutation in RpoB appears to be associated with a fitness cost *in vitro* and reduced virulence *in vivo* (10). In this study, the RpoB and RpoC mutants that appeared at low frequency may have been selected for by FDX, because these mutants were recovered only in FDX-treated patients. However, it is not clear what the clinical implications are of using FDX to treat CDI caused by strains having reduced susceptibility to FDX. In practice, six cases of CDI from whom C. difficile strains with reduced susceptibilities to FDX were isolated were deemed to be clinically cured at the end of treatment with FDX (Table 4). Despite infection with strains having reduced susceptibilities to FDX, clinical response appears to reflect the high concentrations of FDX in stool (mean, 1,225 mg/kg) achieved with a standard regimen of FDX (6).

A potential limitation of this study was that C. difficile strains recovered from patients after antibiotic treatment were isolated at different times, where culture of stool was performed after 10 days of antibiotic treatment, but isolates were also included from specimens collected during the follow-up period, i.e., between days 11 and 28. In a previous report, the same C. difficile ribotypes isolated at baseline were also isolated over a long period of time (14 to 56 days) in recurrent CDI cases (31). Therefore, the differences in the timing of sampling for analysis of C. difficile isolates in the present study appear to be acceptable. Another potential limitation was that the mutations not previously reported, RpoB Gln1149Pro and RpoC Arg326Cys, in C. difficile strains with reduced FDX susceptibility were not analyzed by allelic exchange methods, which are often used to examine the fitness cost of such mutations (11). Because RpoB Gln1149Pro and RpoC Arg326Cys mutations were only detected by comparing the core genomes of FDX-susceptible strains with those from strains having reduced susceptibilities to FDX, which were isolated from the same patient, we suggest that the mutations could contribute to the reduced susceptibility of C. difficile to FDX.

In conclusion, our findings showed that no C. difficile strain with reduced FDX susceptibility was isolated from patients with CDI in hospitals nationwide in Japan before FDX administration. However, mutant C. difficile strains with reduced FDX susceptibilities may have been selected for in the gut of patients treated with FDX. Future studies should assess the potential emergence of CDI caused by C. difficile strains having reduced susceptibility to FDX after the widespread introduction of FDX as a treatment for CDI in Japan.

## MATERIALS AND METHODS

### Ethics.

Procedures completed at LSI Medience Corporation were conducted according to Astellas Research Ethics Committee (AREC) standards established at Astellas Pharma, Inc. Procedures completed at Toho University were deemed to be beyond the scope of examination by the AREC. The study was conducted with approval from the institutional review board of the Toho University Omori Medical Centre (no. 2810-CL-3002). Written informed consent was obtained from patients prior to the start of any study-related procedures using the written information for patients and informed consent form that was approved by the institutional review board of each study site.

### Summary of the clinical trial.

A phase III, VCM-controlled, double-blind, parallel-group study of FDX was conducted in 82 hospitals in Japan between June 2014 and September 2016 (32). The study was registered at ClinicalTrials.gov with the identifier NCT02179658. Briefly, participants were ≥20 years of age with a diagnosis of CDI, defined by the presence of diarrhea (with more than four unformed bowel movements in the 24-h period before randomization) and C. difficile toxin A, B, or both in a stool specimen obtained within 96 h before randomization. Patients could have received up to four doses of MNZ or VCM before randomization but no other potentially effective concurrent treatment for CDI. Enrolled patients were randomized to receive either oral FDX (200 mg every 12 h with intervening placebo given 6 h after FDX) or oral VCM at the clinically recommended dose (33) (125 mg every 6 h with intervening placebo given 6 h after VCM) for 10 days. Patients were assessed every day during the 10-day treatment period, for 2 days afterwards, and at least weekly during the 28-day follow-up. Patients were assessed at an end-of-treatment visit for clinical cure and at an end-of-study visit when recurrence had not been reported.

### Bacterial isolation and species identification.

Stool samples collected within 96 h before study randomization, within 24 h after completing treatment with FDX or VCM (days 10 and 11), and within 28 days (±3 days) after completing treatment with FDX or VCM (days 11 to 31) were sent to a central laboratory (LSI Medience Corporation) and plated directly onto chromID C. difficile agar (bioMérieux, France). Cultures were incubated in an anaerobic chamber (5% CO_2_, 10% H_2_, and 85% N_2_) at 35 ± 2°C for 24 h. Identification of bacterial species was performed using Rapid ID 32A API system (bioMérieux). Frozen, stored C. difficile isolates were sent to the Department of Microbiology and Infectious Diseases at the Toho University School of Medicine.

### Whole-genome sequencing and data analysis.

To determine the draft whole-genome sequence of C. difficile isolates, bacterial DNA was extracted using a standard achromopeptidase and phenol-chloroform method (34). We used the Nextera XT DNA library preparation kit (Illumina, Inc., CA, USA) to prepare DNA libraries for sequencing. Libraries were sequenced on the MiSeq system with the MiSeq reagent v3 (600-cycle) kit (300-bp paired-end reads; Illumina, Inc.). Draft genomes (contigs) were assembled using the CLC Genomics Workbench software (version 11; Qiagen Bioinformatics). Identification and alignment of the following genes were performed using BLASTn (35) and Jalview (version 2) (36): *tpi,* for species identification of C. difficile; *tcdA,* encoding toxin A (TcdA); *tcdB,* encoding TcdB; *cdtA,* encoding binary toxin A (CdtA); *cdtB,* encoding binary toxin B (CdtB); *tcdC,* encoding the negative regulator of the *tcdA* and *tcdB* genes; *gyrA,* encoding DNA gyrase subunit A (GyrA), and *gyrB,* encoding DNA gyrase subunit B (GyrB) for analysis of quinolone resistance-determining regions; and *rpoA*, *rpoB*, and *rpoC,* encoding the respective RpoA, RpoB, and RpoC for analysis of the mechanism of reduced susceptibility to FDX. Genetic variations in the C. difficile toxin gene sequences were identified in BLASTn databases, including C. difficile strain 630 (*tcdA*^+^ *tcdB*^+^ *cdtA-cdtB*^−^, GenBank accession no. NC_009089) and C. difficile strain CD196 (*tcdA*^+^ *tcdB*^+^ *cdtA-cdtB*^+^, GenBank accession no. NC_013315) (27). MLST was performed using C. difficile MLST databases in PubMLST.org (https://pubmlst.org/cdifficile/) and the Center for Genomic Epidemiology MLST 2.0 Web tool (https://cge.cbs.dtu.dk/services/MLST/). Acquired antibiotic resistance genes were identified using the Center for Genomic Epidemiology ResFinder version 2.1 database (https://cge.cbs.dtu.dk/services/ResFinder/).

### Core-genome single-nucleotide polymorphism analysis.

Core-genome SNP-based phylogenetic analysis was performed with whole-genome sequencing data. MiSeq sequencing data were aligned to the genomic sequence of the reference isolate, C. difficile 630, using the Burrows-Wheeler Aligner with ‘SW’ algorithm (37). We aligned the core-genome sequences using the Sequence Alignment/Map software (SAMtools mpileup, version 1.1) (38), which were read using VarScan (version 2.3.7) mpileup2cns (39), and a maximum likelihood phylogenetic tree was constructed using PhyML (40). Using this as the starting tree, we inferred homologous recombination events that imported DNA fragments from beyond the phylogenetic clade and constructed a clonal phylogeny with corrected branch lengths using ClonalFrameML (41). The core genome, excluding homologous recombination sequences estimated using ClonalFrameML, was subjected to SNP detection.

### Antibiotic susceptibility testing.

Antibiotic susceptibility testing was performed at LSI Medience Corporation using the agar dilution method according to Clinical and Laboratory Standards Institute (CLSI) M11-A8 guidelines (42). The antibiotic agents used were FDX, MNZ, VCM, MFLX, and CLDM. The resistance breakpoints for FDX have not been established, while the breakpoints for the remaining drugs were based on CLSI M100-ED28 (MNZ, ≥32 mg/liter; MFLX, ≥8 mg/liter; CLDM, ≥8 mg/liter) (43) and European Committee on Antimicrobial Susceptibility Testing clinical breakpoints version 8.1 (VCM, ≥4 mg/liter) (44). C. difficile ATCC 700057 was used for a susceptibility testing quality control.

### Data availability.

The BioProject no. of this study is PRJDB7714. Draft genome sequences were deposited at the DNA Data Bank of Japan (https://www.ddbj.nig.ac.jp/ddbj/index-e.html) and with GenBank accession numbers BIMY01000000 to BIXW01000000 (see Data Set S1).

## Supplementary Material

Supplemental file 1

Supplemental file 2
