# Supplementary material for: Antimicrobial Susceptibility and Molecular Characterization Using Whole-Genome Sequencing of Clostridioides difficile Collected in 82 Hospitals in Japan between 2014 and 2016
Source: Antimicrob Agents Chemother. 2019 Nov 21;63(12):e01259-19. doi: 10.1128/AAC.01259-19 (PMC6879216; doi:10.1128/AAC.01259-19)
Supplement: Supplemental file 1 [file AAC.01259-19-s0001.pdf]

Table S1. Amino acid substitutions detected in the protein containing antimicrobial resistance factor and possess *erm(B)* gene of first strain of *C. difficile* isolated from CDI participants

			RpoA[a]				RpoB[b]												RpoC[c]							GyrA[d]		GyrB[e]		GyrA or GyrB		MLS[f] resistant gene
ST	No. of strains		Gly91Asp	Ser94Ile	Pro115Ser		Asp492Val	His502Asn	Arg505Lys	Ile548Met	Ile750Met/Val	Glu1037Gln	Asp1160Glu	Ala1205Val	Asn1207Ala	Ala1208Thr	Asp1232Glu		Thr543Ile	Asn564Lys	Ala617Ser	Ile788Val	Ile833Leu	Pro1084Thr		Thr82Ile/Val		Asp426Ala/Asn/Val		Thr82Ile/Val or Asp426Ala/Asn/Val		*erm(B)*
ST17	61		0	0	0		0	2	2	0	0	0	0	0	0	0	0		0	0	0	0	0	0		60		6		61		34
ST8	26		0	0	0		0	0	0	0	0	0	0	0	0	0	0		0	0	0	0	0	0		17		2		18		6
ST2	21		0	0	0		0	0	0	0	21	0	0	0	0	0	0		0	0	0	0	0	0		1		0		1		0
ST81	19		0	0	0		0	0	0	0	19	0	0	0	0	0	0		0	0	0	0	0	0		7		19		19		12
ST183	13		0	0	0		1	0	0	1	1	0	0	0	0	0	0		0	0	0	0	0	0		13		13		13		6

ST55	5		0	0	0		0	0	0	0	0	0	0	0	0	0	0		0	0	0	0	0	0		0		0		0		0
ST37	4		0	0	0		0	0	0	0	4	0	0	0	0	0	0		0	0	0	0	0	0		0		0		0		4
ST5	3		0	3	0		0	0	0	0	0	0	0	0	0	0	0		0	0	0	0	0	0		0		0		0		0
ST14	3		0	0	0		0	0	0	0	0	0	0	0	0	0	0		0	0	0	0	0	0		0		0		0		0
ST15	3		0	0	0		0	0	0	0	0	0	0	0	0	0	0		0	0	0	0	0	0		0		0		0		1
Other STs[g]	30		3	0	1		0	1	1	0	4	2	1	2	2	2	2		2	1	1	1	4	1		5		1		5		4
Total	188		3	3	1		1	3	3	1	49	2	1	2	2	2	2		2	1	1	1	4	1		103		41		117[h]		67

[a]RpoA, DNA-directed RNA polymerase subunit alpha encoded by *rpoA* is target of fidaxomicin;

[b]RpoB, DNA-directed RNA polymerase subunit beta encoded by *rpoB* is target of fidaxomicin;

[c]RpoC, DNA-directed RNA polymerase subunit beta' encoded by *rpoC* is target of fidaxomicin;

[d]GyrA, DNA gyrase subunit A is target of the quinolones, displayed only the amino acid substitution of QRDRs;

[e]GyrB, DNA gyrase subunit B is target of the quinolones, displayed only the amino acid substitution of QRDRs;

[f]MLS_B, macrolide, lincosamide, streptogramin B;

[g]Other STs, for ST with less than two strains, ST1, ST3, ST6, ST11, ST13, ST27, ST35, ST42, ST47, ST48, ST53, ST54, ST58, ST59, ST67, ST82, ST98, ST100, ST103, ST109, ST182, ST470

[h]Three MXFX resisntant strain were not found Thr82Ile/Val or Asp426Ala/Asn/Val.
